# Supplementary material for: The Atr-Chek1 pathway inhibits axon regeneration in response to Piezo-dependent mechanosensation
Source: Nat Commun. 2021 Jun 22;12:3845. doi: 10.1038/s41467-021-24131-7 (PMC8219705; doi:10.1038/s41467-021-24131-7)
Supplement: Supplementary file 1 — Supplementary information [file 41467_2021_24131_MOESM1_ESM.pdf]

The Atr-Chek1 pathway inhibits axon regeneration in response to Piezo-dependent mechanosensation

SUPPLEMENTARY INFORMATION

Supplementary Figures 1-11

Supplementary Table

Supplementary Movies 1-6

Supplementary Figure 1

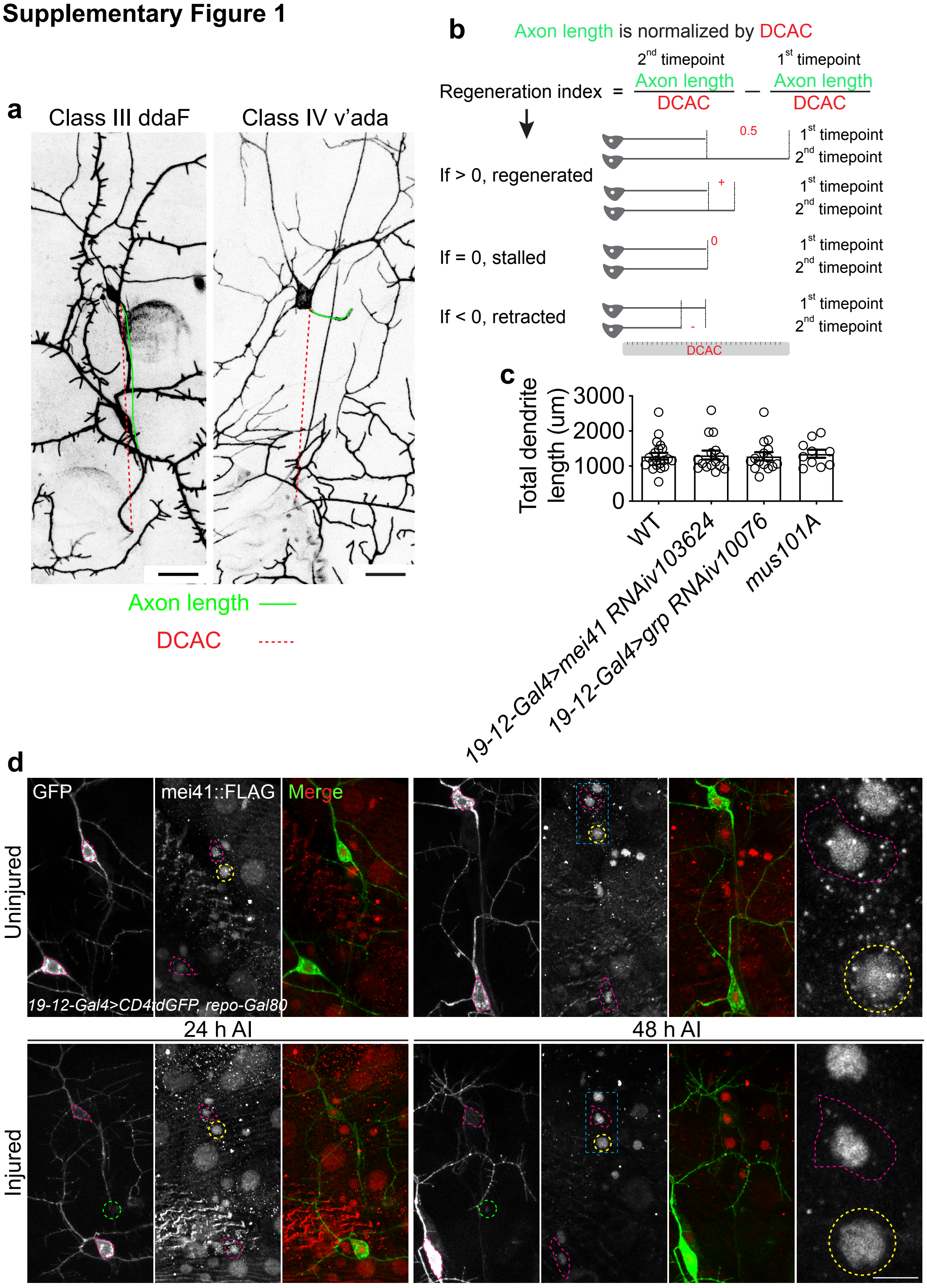

Supplementary Figure 1. Quantification of sensory axon regeneration in the fly PNS, dendrite branching and mei41 expression.

(**a**) "Regeneration index" is calculated as an increase of "axon length"/"distance between the cell body and the axon converging point (DCAC)". (**b**) A schematic showing the axon regeneration extent: regenerated, stalled or retracted. (**c**) The dendrites of class III da neurons of various genotypes were traced at 72 h AI and the total dendrite length was quantified. *N* = 20, 15, 15 and 10 neurons from 3 to 5 larvae. Data are presented as mean values +/- SEM. (**d**) Expression and localization of mei41 with and without injury. Mei41 is present mainly in the nucleus of class III da sensory neurons. No significant difference is observed after axon injury. The injury site is demarcated by the green dashed circle. Class III and IV da neurons are outlined by the red and yellow dashed lines, respectively. *N* = 8 segments from 4 larvae. No statistical difference is detected by One-way ANOVA followed by Dunnett's test (b). Source data are provided as a Source Data file.

Supplementary Figure 2

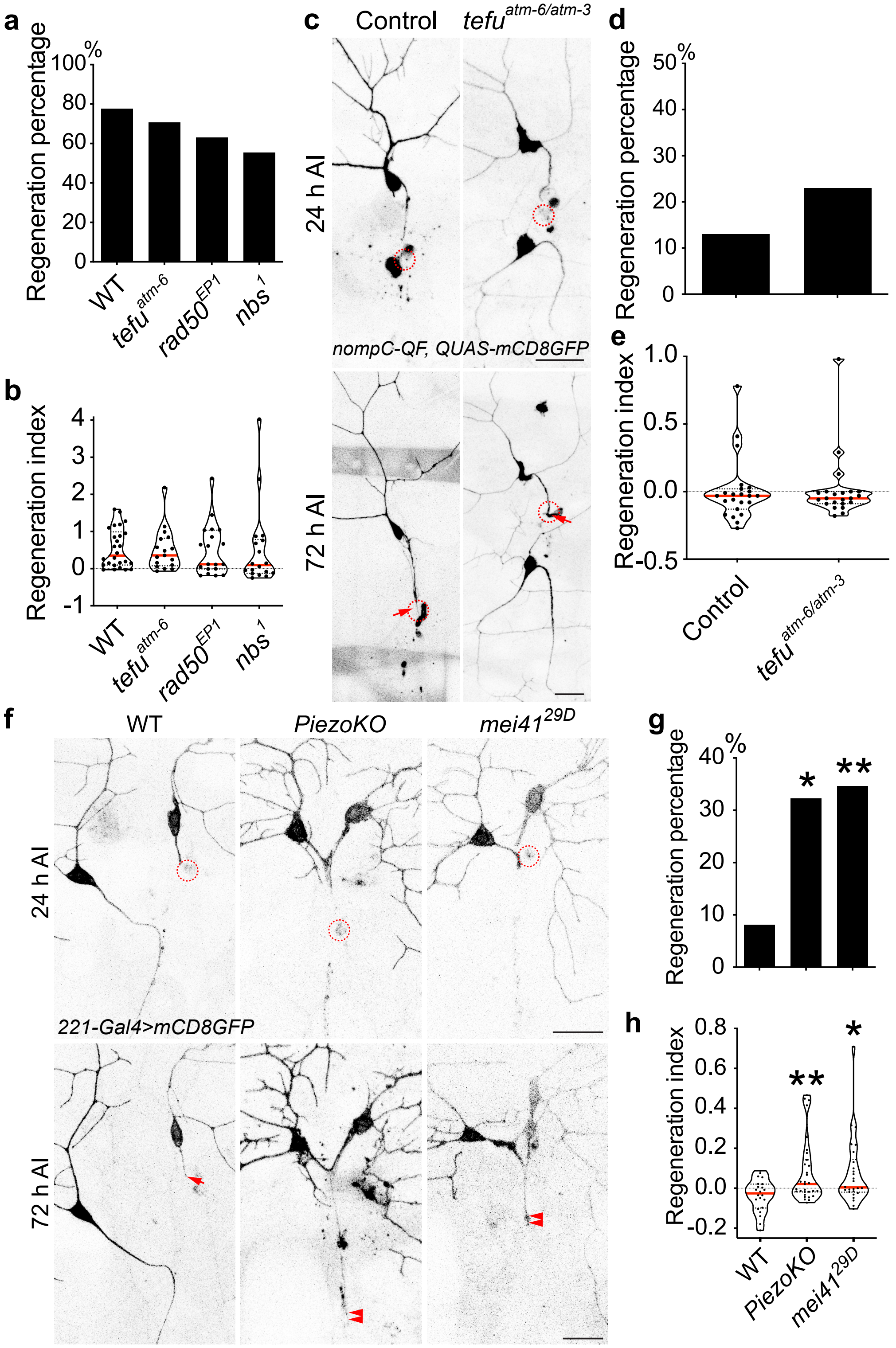

Supplementary Figure 2. The Atm/tefu pathway does not regulate axon regeneration in da sensory neurons in flies, and class I da neuron axon regeneration.

(**a**, **b**) Inhibiting the Atm/tefu pathway does not significantly alter class IV da neuron axon regeneration. Quantifications of class IV da neuron axon regeneration with Regeneration percentage (a) and Regeneration index (b). *N* = 27, 17, 19 and 18 neurons from 5 to 7 larvae. (**c** to **e**) Atm/tefu loss of function does not promote class III da neuron axon regeneration. (c) Atm/tefu removal as in $tefu^{atm\text{-}6/atm\text{-}3}$ mutants does not increase axon regeneration. The injury site is demarcated by the dashed circle. Arrow marks axon stalling. Scale bar = 20 µm. Quantifications of class III da neuron axon regeneration with Regeneration percentage (d) and Regeneration index (e), shown in the scatter plot. *N* = 23 and 22 neurons from 7 to 8 larvae. No statistical difference is detected by two-sided Fisher's exact test (a and d), one-way ANOVA followed by Dunnett's test (b), two-tailed unpaired Student's t-test (e). (**f** to **h**) *PiezoKO* and $mei41^{29D}$ mutants enhance class I da neuron axon regeneration. The injury site is demarcated by the dashed circle. Arrowheads mark regenerating axons. Scale bar = 20 µm. Quantifications of class I da neuron axon regeneration with Regeneration percentage (g) and Regeneration index (h). *N* = 40, 31 and 26 neurons from 7 to 11 larvae. P = 0.012, 0.0083. *$P < 0.05$, **$P < 0.01$ by two-sided Fisher's exact test (g), one-way ANOVA followed by Dunnett's test (h). Source data are provided as a Source Data file.

Supplementary Figure 3

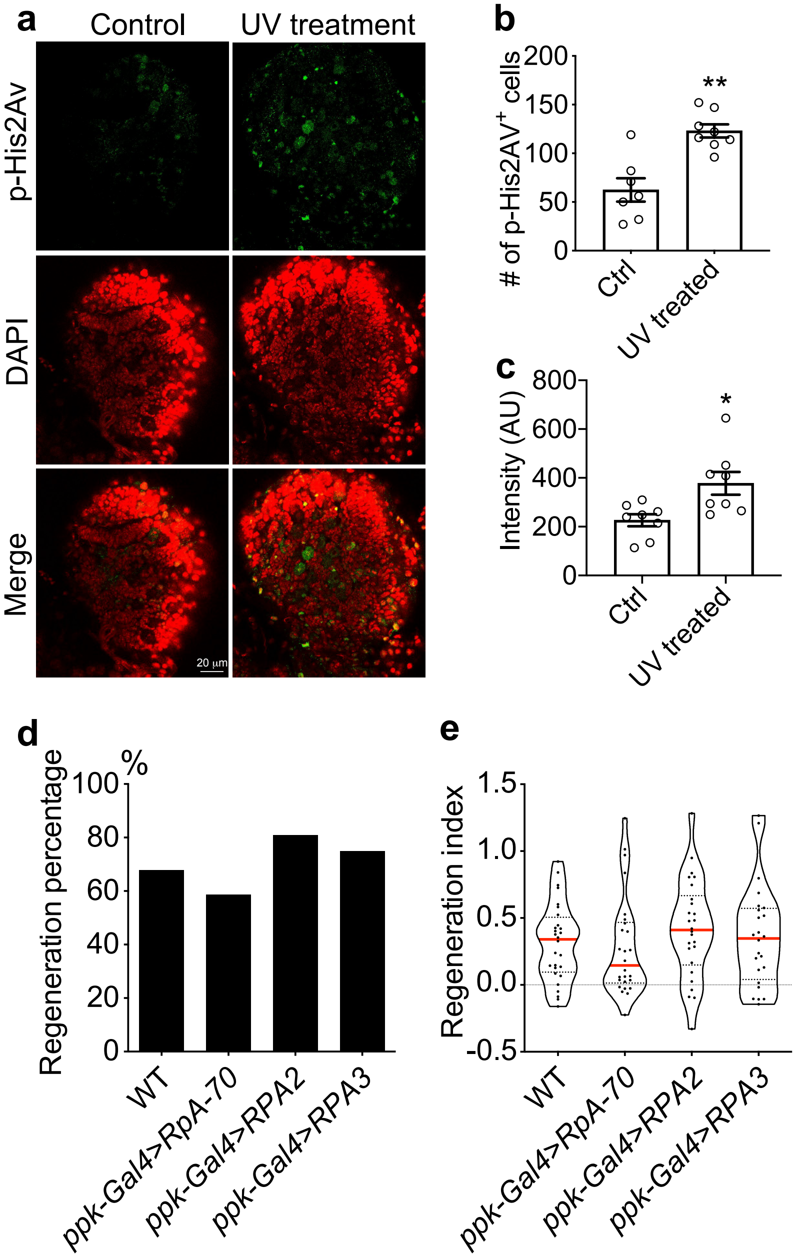

Supplementary Figure 3. P-His2Av staining after UV treatment, and overexpression of RPAs does not reduce axon regeneration.

(**a-c**) P-His2Av staining positive control, and UV treatment increases p-His2Av staining in the larval brain. (a) Images show p-His2Av antibody and DAPI staining of larval brain lobes at 48 hours after UV treatment (from the ventral side of the larva). Larvae were collected at 48 hours after egg laying (h AEL) and the control group is with sham treatment. Scale bar = 20 μm. (b) Quantifications of the number of p-His2Av$^+$ cells in the ventral hemisphere of each brain lobe. N = 7 brain lobes for Ctrl and 8 for UV treatment. $P = 0.0015$. (c) Quantifications of the intensity of each brain lobe (N = 8 brain lobes for Ctrl and 8 for UV treatment. Mean intensity from 10-15 representative cells is shown. $P = 0.0157$. Data are presented as mean values +/- SEM. (**d**, **e**) Quantifications of class IV da neuron axon regeneration with Regeneration percentage (d) and Regeneration index (e). N = 31, 29, 26 and 24 neurons from 7 to 8 larvae. *$P < 0.05$, **$P < 0.01$ by two-tailed unpaired Student's t-test (b and c), two-sided Fisher's exact test (d), one-way ANOVA followed by Dunnett's test (e). Source data are provided as a Source Data file.

Supplementary Figure 4

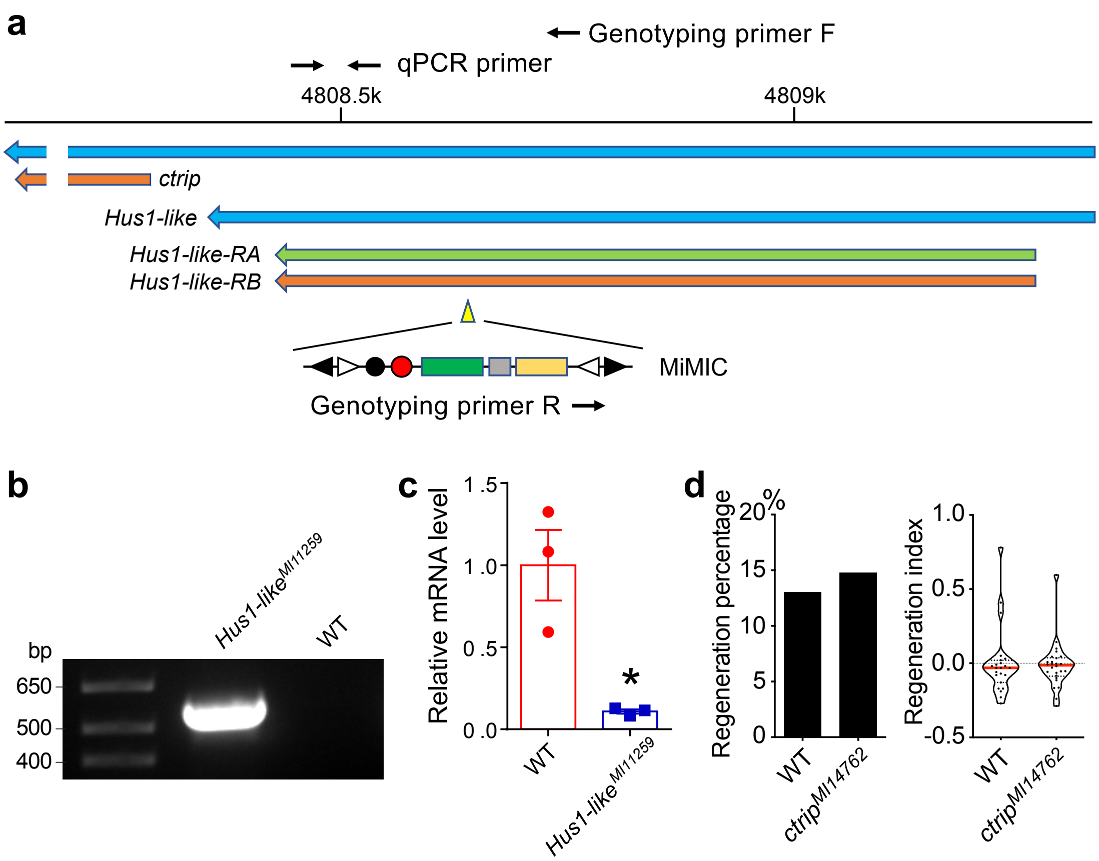

Supplementary Figure 4. The insertional allele of Hus1-like – *Hus1-like*^{MI11259} is a loss of function mutant, and *ctrip* mutants do not show increased axon regeneration.

(**a**) The *Hus1-like*^{MI11259} insertional locus, and primers for genotyping and quantitative RT-PCR. MiMIC: *Minos*-mediated integration cassette. (**b**) Genomic PCR using the genotyping primers confirms the insertion. (**c**) Quantitative RT-PCR shows a significant reduction of the *Hus1-like* transcripts in the *Hus1-like*^{MI11259} mutants. *rp49* was used as the loading control, and the *Hus1-like* mRNA level was normalized to that of WT. $N = 3$ biological replicates from 5-10 larvae each. $P = 0.0145$. Data are presented as mean values +/- SEM. *$P < 0.05$, by two-tailed unpaired Student's t-test. (**d**) Quantifications of class III da neuron axon regeneration with Regeneration percentage and Regeneration index. $N = 23$ and 27 neurons from 6 to 7 larvae. No statistical difference is detected by two-sided Fisher's exact test or two-tailed unpaired Student's t-test. Source data are provided as a Source Data file.

Supplementary Figure 5

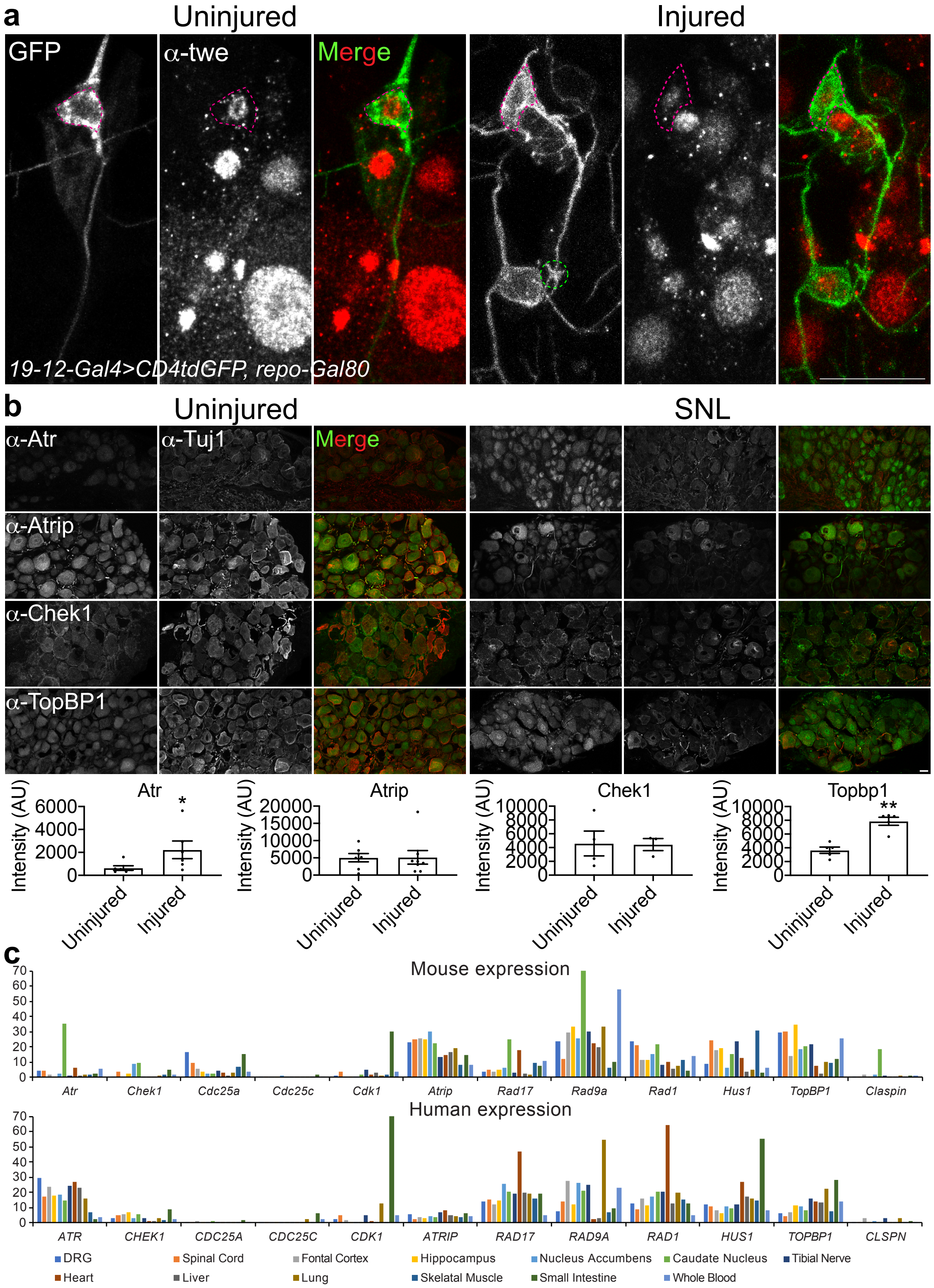

Supplementary Figure 5. The expression pattern of Atr pathway members in fly, mouse and human.

(**a**) Expression and localization of twe with or without injury. Twe is present mainly in the nucleus of class III da sensory neurons. No significant difference is observed after axon injury. The injury site is demarcated by the green dashed circle. Class III da neuron cell bodies are outlined by the red dashed circle. Twe is also expressed in other da neurons. Scale bar = 20 μm. (**b**) Immunostaining for Atr, Atrip, Chek1 and TopBP1 using mouse DRG tissue sections. All four proteins are expressed with or without sciatic nerve lesion (SNL) and their expression levels were quantified. DRG neurons were counterstained with the α-Tuj1 antibody. N = 6, 6 (for Atr), 7, 8 (for Atrip), 4, 3 (for Chek1), 5, 5 (for Topbp1) tissue sections. P = 0.0411, 0.4634, 0.6286, 0.0079. Data are presented as mean values +/- SEM. Scale bar = 20 μm. (**c**) Expression level of Atr pathway members in various tissues in mouse and human. All of them are expressed in the mouse and human DRG, although at a low to medium level. $^*P < 0.05$, $^{**}P < 0.01$ by two-tailed unpaired Student's t-test (b). Source data are provided as a Source Data file.

Supplementary Figure 6

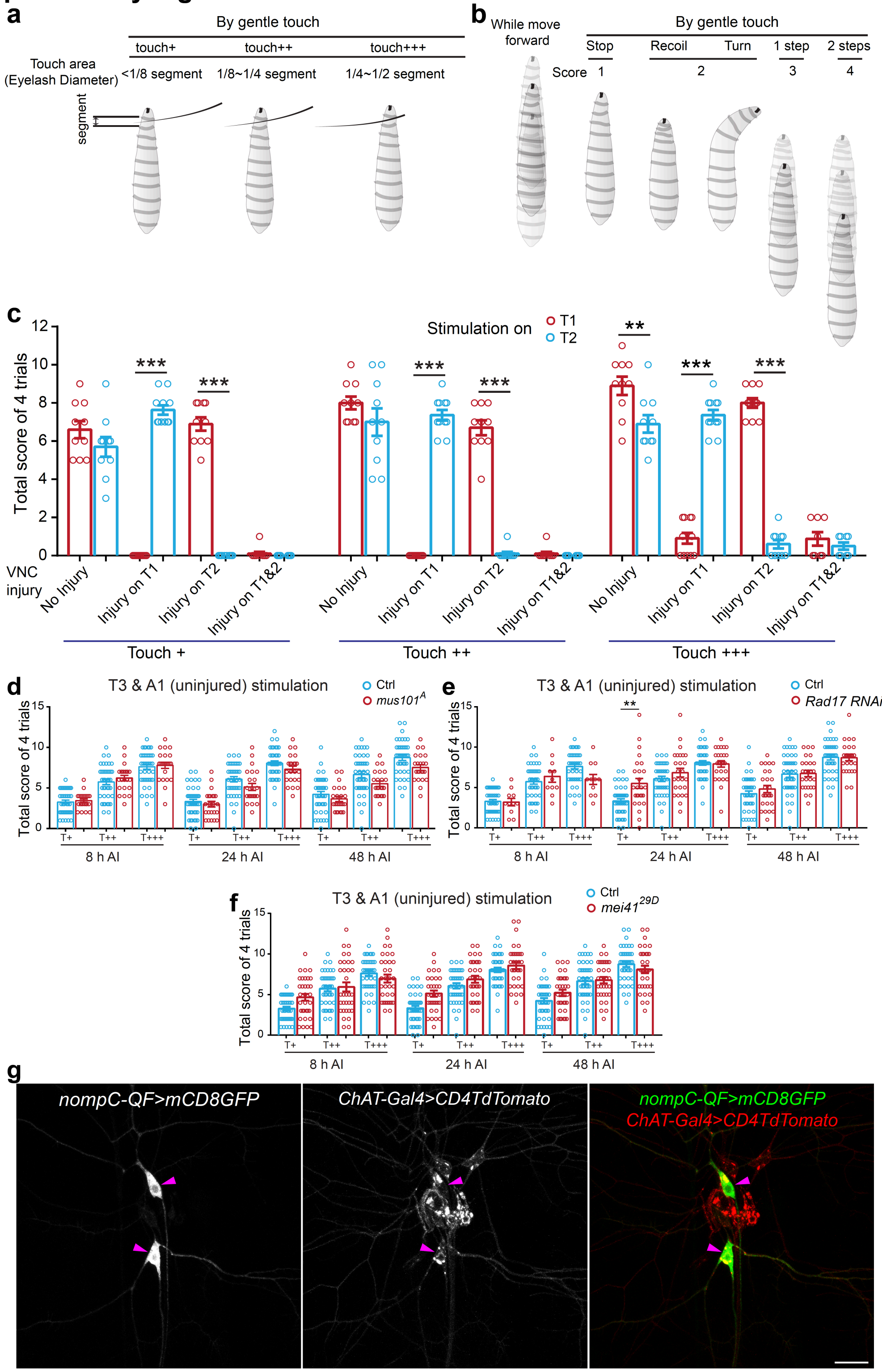

Supplementary Figure 6. The modified gentle-touch behavioral paradigm for assessing functional recovery after CNS injury in flies.

(**a**) The definition of the gentle-touch stimulus. The intensity of the stimulation is subcategorized based on the contact area between the eyelash and the body segment: touch+, touch++ and touch+++, with the eyelash diameter covering <1/8, 1/8-1/4 or 1/4-1/2 of the segment, respectively. (**b**) The scoring system for the gentle-touch response – gently touching larval anterior segments with an eyelash elicits a set of stereotypical responses. While the larvae are moving forward, by a gentle touch, if they stop (hesitate), score 1; recoil or turn, score 2; one step back (single reverse contractile wave), score 3; two or more steps back (multiple waves of reverse contraction), score 4; no response, score 0. (**c**) Injuring the class III da neuron axon bundle at T1 or T2 in the VNC leads to impaired touch response specifically at segment T1 or T2, without affecting neighboring segments. Total response scores from 4 trials are added and shown in scatter plots. N = 10 (no injury), 11 (injury on T1), 10 (injury on T2), 8 (injury on both T1 and T2) larvae. (**d** to **f**) The behavioral response in the uninjured T3 and A1 segments are comparable among the different genotypes – *mus101*^{A} mutants (d), class III da neuron specific *Rad17* RNAi (e) and *mei41*^{29D} mutants (f). Larvae also show a graded response according to the stimulation intensity. N = 41 larvae for Ctrl, 23 for *mus101*^{A}, 33 for *mei41*^{29D}, 11, 26 and 26 for *Rad17 RNAi* at 8 h, 24 h and 48 h. Data are presented as mean values +/- SEM. *$P < 0.05$, **$P < 0.01$, ***$P < 0.001$ by one-way ANOVA followed by Tukey's test (c to f). (**g**) Class III da neurons are co-labeled by *nompC-QF>mCD8GFP* (the class III da neuron marker) and *ChAT-Gal4>CD4TdTomato*. Arrowhead marks the class III da neuron cell body. Scale bar = 20 µm. Source data are provided as a Source Data file.

Supplementary Figure 7

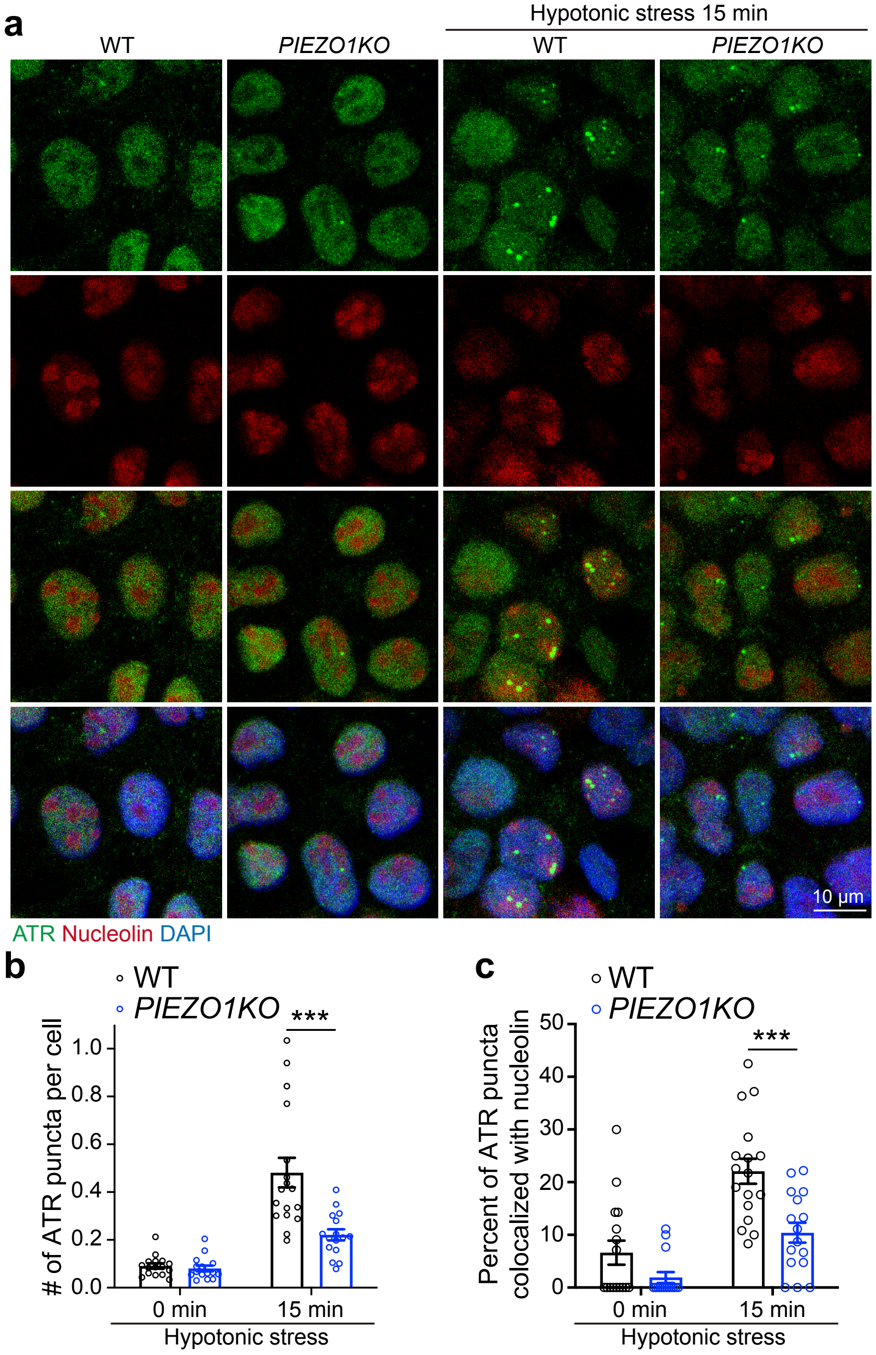

Supplementary Figure 7. Endogenous ATR clustering induced by hypotonic stress. (**a**) Hypotonic stress induces endogenous ATR clustering in WT HEK 293T cells, which are colocalized with nucleolin. The clustering is reduced in *PIEZO1KO*. (**b**) Quantification of the number of ATR clusters per cell. (**c**) Quantification of the percentage of ATR puncta colocalized with nucleolin. N = 16, 17 (for 0 min), 15, 16 (for 15 min) fields of view. Data are presented as mean values +/- SEM. ***P < 0.001 by Two-way ANOVA followed by Sidak's multiple comparisons test. Source data are provided as a Source Data file.

Supplementary Figure 8

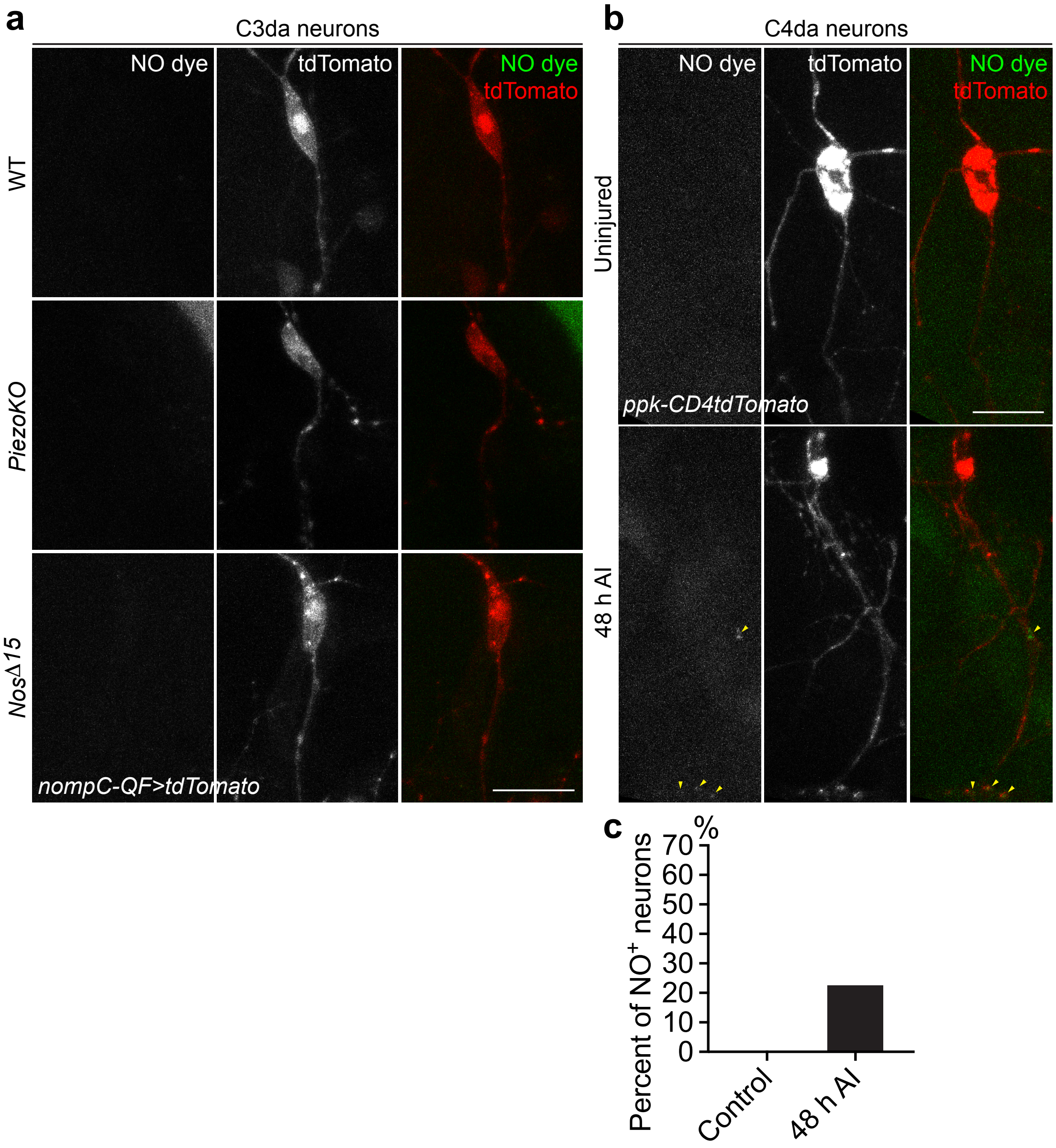

Supplementary Figure 8. NO imaging in uninjured class III, uninjured and injured class IV da neurons. (**a**) No obvious NO fluorescence signal is detected in uninjured class III da neurons among WT, *PiezoKO* and $Nos^{\Delta 15}$ mutants. *N* = 8, 12 and 8 neurons from 3 to 4 larvae. (**b**, **c**) NO fluorescence signal is detected in injured class IV da neurons, but to a lesser extent than class III da neurons. Arrowheads indicate NO staining. *N* = 8, 13 neurons from 3 to 4 larvae. Scale bar = 20 μm. Source data are provided as a Source Data file.

Supplementary Figure 9

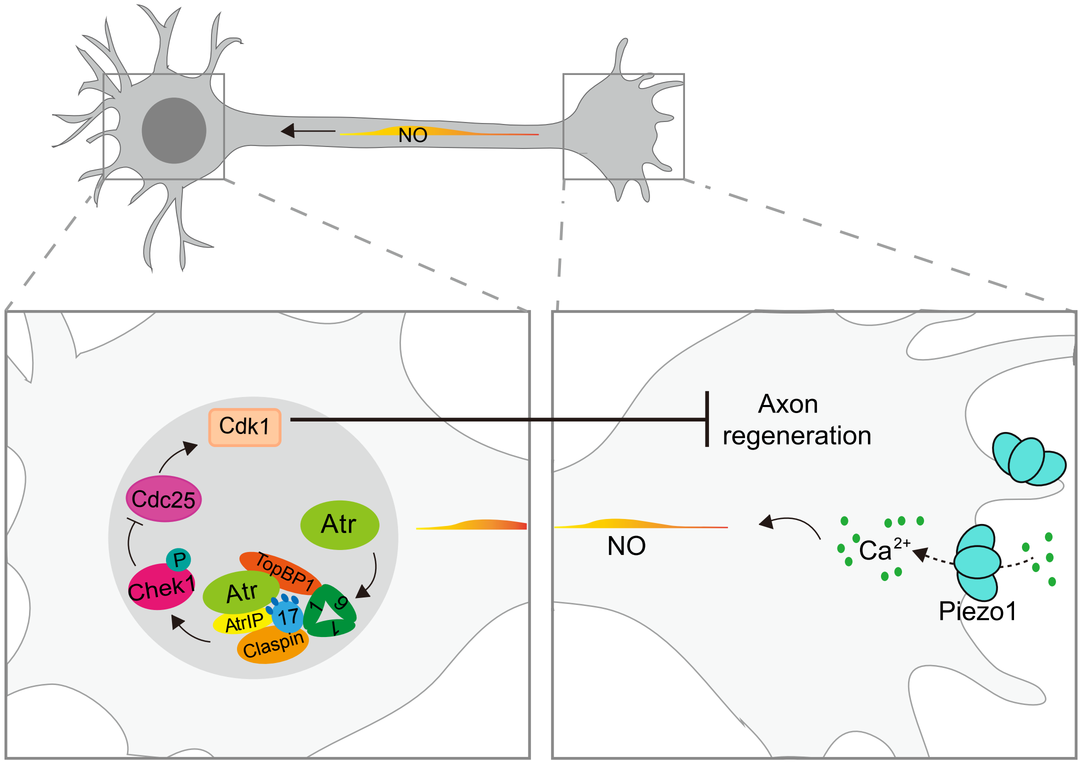

Supplementary Figure 9. The proposed Piezo-Nos-Atr-Chek1 pathway in inhibiting axon regeneration. The proposed Piezo-Nos-Atr-Chek1 signaling cascade that inhibits axon regeneration. During axon regeneration, the mechanical force resulting from the interactions between the growth cone and the environment, activates the mechanosensitive ion channel Piezo at the growth cone tip, leading to local calcium influx and activation of Nos, which then produces NO. NO functions as a second messenger and propagates to the nucleus where it activates Atr and the associated checkpoint complex. Atr then phosphorylates and activates Chek1, which phosphorylates and inactivates Cdc25, inhibiting its ability to dephosphorylate and activate Cdk1. The phosphorylated and inactive Cdk1 suppresses axon regeneration through downstream effectors.

Supplementary Figure 10

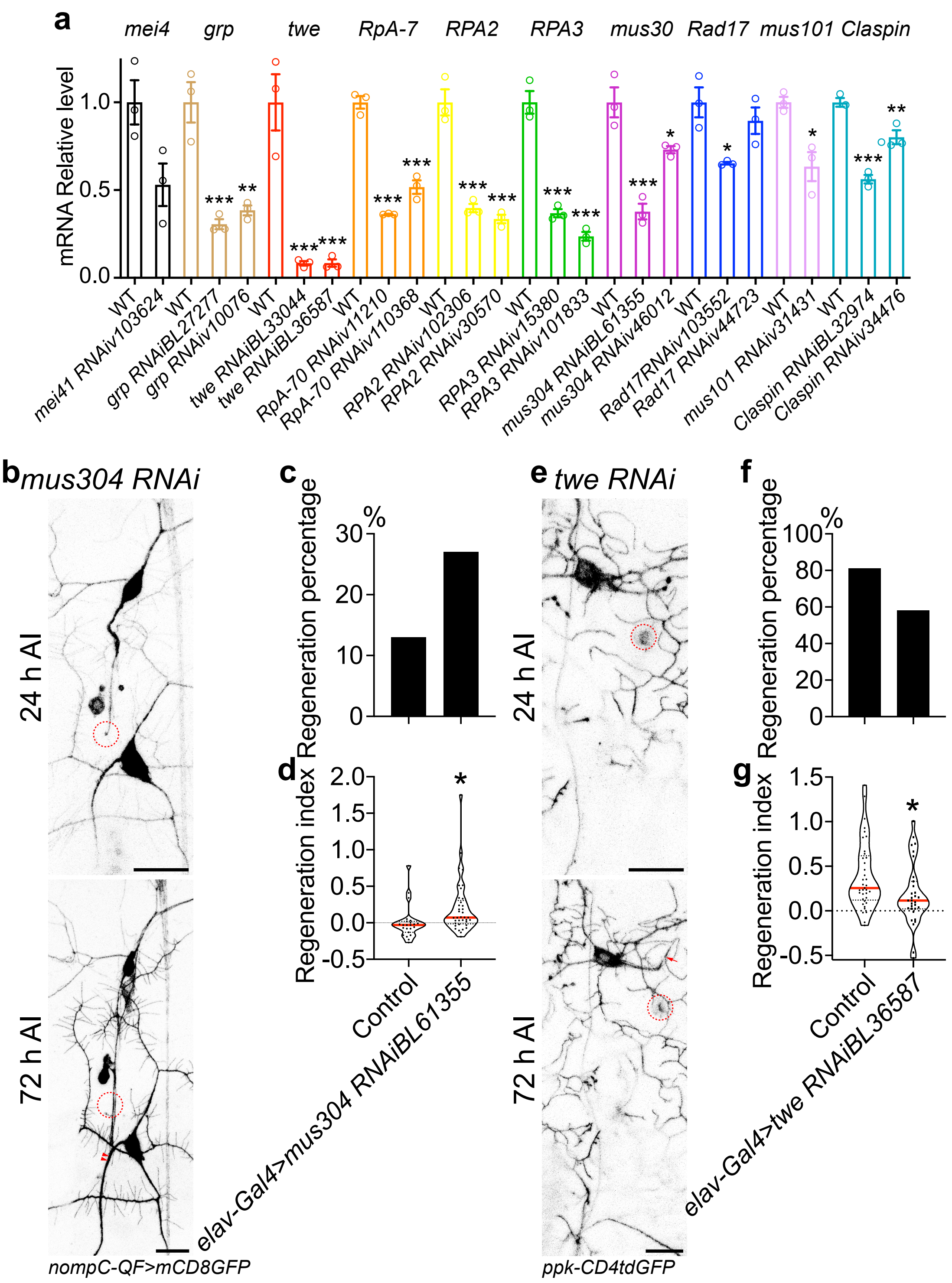

Supplementary Figure 10. Confirmation of RNAi knockdown efficiency. (**a**) Q-PCR analyses of the mRNA transcripts after RNAi expression using the pan-neuronal *elav-Gal4*. $N = 3$ biological replicates. Data are presented as mean values +/- SEM. (**b**) Knocking down mus304 using the pan-neuronal *elav-Gal4* increases class III da neuron axon regeneration. (**c**, **d**) Quantifications of class III da neuron axon regeneration with Regeneration percentage (c) and Regeneration index (d). $N = 23$ and 37 neurons from 6 to 10 larvae. $P = 0.0114$. (**e**) Knocking down twe using the pan-neuronal *elav-Gal4* decreases class IV da neuron axon regeneration. The injury site is demarcated by the dashed circle. Arrow marks axon stalling while arrowheads show the regrowing axon tips. Scale bar = 20 μm. (**f**, **g**) Quantifications of class IV da neuron axon regeneration with Regeneration percentage (f) and Regeneration index (g). $N = 37$ and 31 neurons from 5 to 8 larvae. $P = 0.0226$. $^*P < 0.05$ by two-tailed unpaired Student's t-test (d, g). Source data are provided as a Source Data file.

Supplementary Figure 11

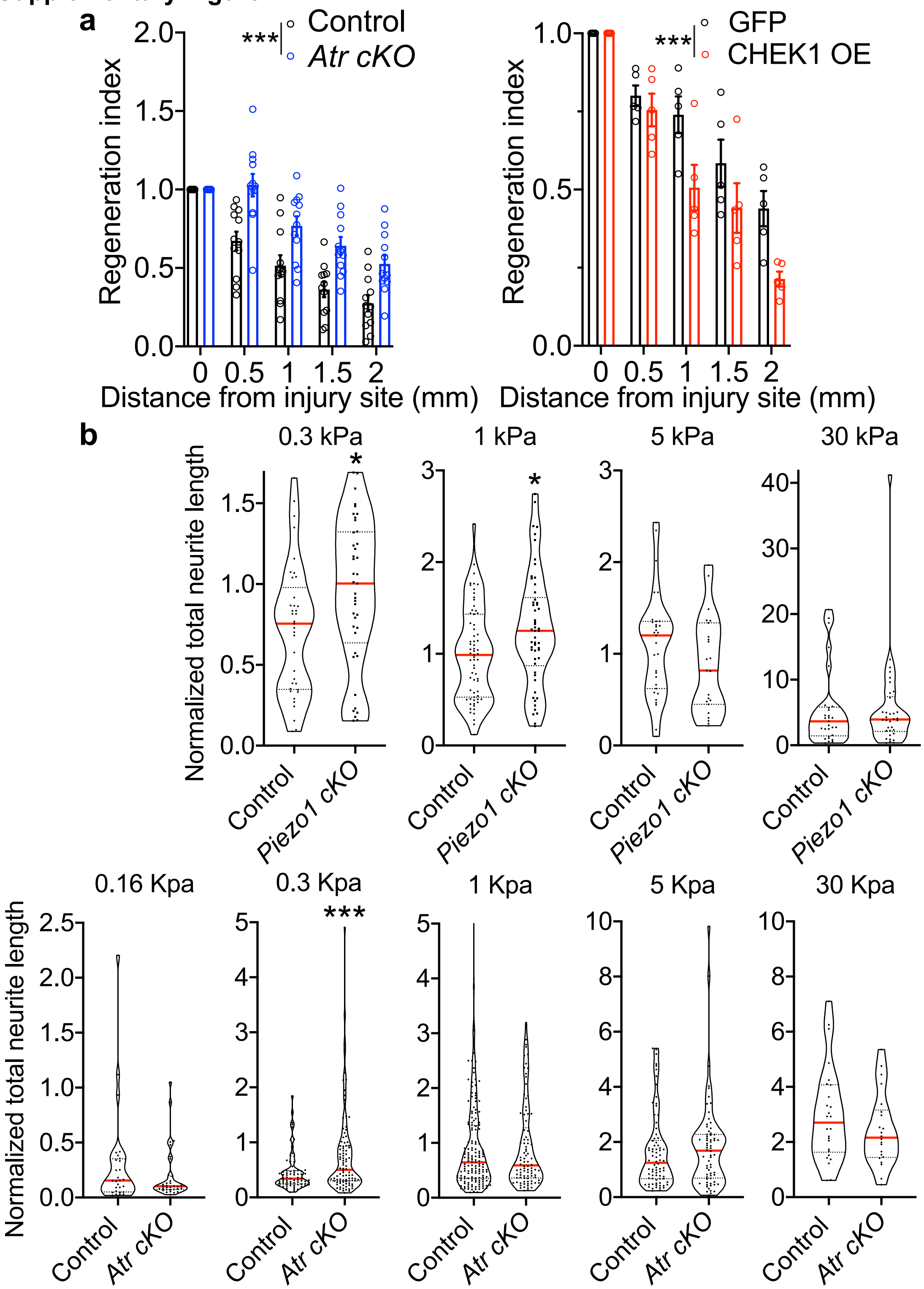

Supplementary Figure 11. Individual data plots for Figure 8. (**a**) The scatter plots of the graphs in Figure 8h and 8j. Data are presented as mean values +/- SEM. (**b**) The violin plots of the bar graphs in Figure 8l and 8m. P = 0.0188, 0.0104, 0.8871, 0.1567, 0.2684, 0.0005, 0.6222, 0.4928, 0.2176. *P < 0.05, ***P < 0.001 by two-tailed unpaired Student's t-test (b), or Two-way ANOVA (a). Source data are provided as a Source Data file.

Supplementary Table 1. Primers.

Hus1-like qPCR	Primer F	AGCACTTCAACTCCCTAACG
	Primer R	CCACATCCTGTCGTACATCG
rp49 qPCR	Primer F	CAGTCGGATCGATATGCTAAGCTG
	Primer R	TAACCGATGTTGGGCATCAGATAC
Hus1-like genotyping	Primer F	GAAGTGGTGCACGATGTTCCAG
	Primer R	ACTACTCCCGAAAACCGCTTCT
mus304	Primer F	GCGAGTGCTATGTAAAGTTGTG
	Primer R	TGAAAGACAAGGGACAGAAGG
Rad17	Primer F	TTGTCGAGGACTTTCCCAAC
	Primer R	AGGCGGTAGCTGATATTCAATC
mus101	Primer F	CATCACGGAGCAGAGAACAC
	Primer R	TTCACGAAGTACGCGCAG
Claspin	Primer F	ACCTGCTAAACCGTCTGATG
	Primer R	TTGGTTCTTTTGCGATTTCCTC
RpA-70	Primer F	CAGCACAATGGAGAATTAGAGG
	Primer R	TTTGGACTTAACCTCGGCG
RPA2	Primer F	CAGCAATCAAAAGGGAGAAGG
	Primer R	AACTCGATGTTTCCCTCTGG
RPA3	Primer F	AGTTCCACAGACAACCACAAG
	Primer R	CGCCAAACTCGATGACCTC
twe	Primer F	GAACCCAAAATCTGAACTTCCC
	Primer R	TTGGCCTGGTATTCCATATCG
grp	Primer F	CGTGAAAATATCGGACTTTGGC
	Primer R	GCGTGATATGCCTTCTGTAGC
mei41	Primer F	GACACATTCCTACTCTGGCTG
	Primer R	CTTTGAAGATTGATGCACTCGC
