# Supplementary material for: The Atr-Chek1 pathway inhibits axon regeneration in response to Piezo-dependent mechanosensation
Source: Nat Commun. 2021 Jun 22;12:3845. doi: 10.1038/s41467-021-24131-7 (PMC8219705; doi:10.1038/s41467-021-24131-7)
Supplement: Supplementary file 3 — Description of Additional Supplementary Files [file 41467_2021_24131_MOESM3_ESM.pdf]

Description of Additional Supplementary Files

Supplementary Movie 1
Related to Figure 4 and Supplementary Figure 6. WT 8 h AI

Supplementary Movie 2
Related to Figure 4 and Supplementary Figure 6. WT 48 h AI

Supplementary Movie 3
Related to Figure 4 and Supplementary Figure 6. Mus101A mutants 8 h AI

Supplementary Movie 4
Related to Figures 4 and Supplementary Figure 6. Mus101A mutants 48 h AI

Supplementary Movie 5
Related to Figure 4 and Supplementary Figure 6. Class III da neuron specific Rad17 RNAi 8 h AI

Supplementary Movie 6
Related to Figure 4 and Supplementary Figure 6. Class III da neuron specific Rad17 RNAi 48 h AI
