# Supplementary material for: The Atr-Chek1 pathway inhibits axon regeneration in response to Piezo-dependent mechanosensation
Source: Nat Commun. 2021 Jun 22;12:3845. doi: 10.1038/s41467-021-24131-7 (PMC8219705; doi:10.1038/s41467-021-24131-7)
Supplement: Supplementary file 10 — Reporting Summary [file 41467_2021_24131_MOESM10_ESM.pdf]

nature research

Corresponding author(s): Yuanquan Song

Last updated by author(s): May 16, 2021

Reporting Summary

Nature Research wishes to improve the reproducibility of the work that we publish. This form provides structure for consistency and transparency in reporting. For further information on Nature Research policies, see our Editorial Policies and the Editorial Policy Checklist.

Statistics

For all statistical analyses, confirm that the following items are present in the figure legend, table legend, main text, or Methods section.

n/a	Confirmed	
☐	☒	The exact sample size (n) for each experimental group/condition, given as a discrete number and unit of measurement
☐	☒	A statement on whether measurements were taken from distinct samples or whether the same sample was measured repeatedly
☐	☒	The statistical test(s) used AND whether they are one- or two-sided<br>*Only common tests should be described solely by name; describe more complex techniques in the Methods section.*
☐	☒	A description of all covariates tested
☐	☒	A description of any assumptions or corrections, such as tests of normality and adjustment for multiple comparisons
☐	☒	A full description of the statistical parameters including central tendency (e.g. means) or other basic estimates (e.g. regression coefficient) AND variation (e.g. standard deviation) or associated estimates of uncertainty (e.g. confidence intervals)
☐	☒	For null hypothesis testing, the test statistic (e.g. F, t, r) with confidence intervals, effect sizes, degrees of freedom and P value noted<br>*Give P values as exact values whenever suitable.*
☒	☐	For Bayesian analysis, information on the choice of priors and Markov chain Monte Carlo settings
☒	☐	For hierarchical and complex designs, identification of the appropriate level for tests and full reporting of outcomes
☒	☐	Estimates of effect sizes (e.g. Cohen's d, Pearson's r), indicating how they were calculated

Our web collection on statistics for biologists contains articles on many of the points above.

Software and code

Policy information about availability of computer code

Data collection	Image data collection: Leica LAS X, Zeiss ZEN 2012
Data analysis	Image data analysis: ImageJ 1.52q, Neuronstudio 0.9.92. Statistical analyses: GraphPad Prism 8.

For manuscripts utilizing custom algorithms or software that are central to the research but not yet described in published literature, software must be made available to editors and reviewers. We strongly encourage code deposition in a community repository (e.g. GitHub). See the Nature Research guidelines for submitting code & software for further information.

Data

Policy information about availability of data

All manuscripts must include a data availability statement. This statement should provide the following information, where applicable:

- Accession codes, unique identifiers, or web links for publicly available datasets
- A list of figures that have associated raw data
- A description of any restrictions on data availability

The data generated to support the findings of this study are available from the corresponding author upon reasonable request. The source data for all Figures and Supplementary Figures are provided as a Source Data files.

Field-specific reporting

Life sciences study design

All studies must disclose on these points even when the disclosure is negative.

Sample size	For regeneration experiments in flies, we collected >10 samples for each genotype and condition. For regeneration experiments in mouse, we collected >=5 animals for each condition. For regeneration experiments in cultured cells, we performed >=4 independent experiments. For immunostaininig experiments, we collected >=3 samples for each genotype and condition. The sample size and statistical tests were chosen based on previous studies with similar methodologies (Song et al., 2012; Li et al., 2020; Wen et al., 2017; Wen et al., 2018) and the data met the assumptions for each statistical test performed.
Data exclusions	No data were excluded from the analyses.
Replication	Each experiment was successfully reproduced at least three times and was performed on multiple days.
Randomization	All data were collected and processed randomly.
Blinding	The group allocation in data collection can not be blinded due to the needs of the experiments. Specific genotypes and conditions are collected for data analyses. The data quantifications were performed blindly by co-authors.

Reporting for specific materials, systems and methods

We require information from authors about some types of materials, experimental systems and methods used in many studies. Here, indicate whether each material, system or method listed is relevant to your study. If you are not sure if a list item applies to your research, read the appropriate section before selecting a response.

Materials & experimental systems

n/a	Involved in the study	
☐	☒	Antibodies
☐	☒	Eukaryotic cell lines
☒	☐	Palaeontology and archaeology
☐	☒	Animals and other organisms
☒	☐	Human research participants
☒	☐	Clinical data
☒	☐	Dual use research of concern

Methods

n/a	Involved in the study	
☒	☐	ChIP-seq
☒	☐	Flow cytometry
☒	☐	MRI-based neuroimaging

Antibodies

Antibodies used	Mouse anti-p-His2Av antiserum (UNC93-5.2.1, 0.3 ug/mL, Developmental Studies Hybridoma Bank), mouse Anti-FLAG M2 (F3165, 1:500, Sigma), rabbit anti-twe (1:100, O'Farrell Lab), mouse anti-Tuj1 (801202, 1:5000, BioLegend), rabbit anti-ATR (13934, 1:500, Cell Signaling), mouse anti-Nucleolin (ab136649, 1:1000, Abcam), rabbit anti-Atrip (PA1-519, 1:400, ThermoFisher), rabbit anti-Chek1 (AV32589, 1:100, Sigma), rabbit anti-TopBP1 (LS-C663420-20, 1:1000, LSBio), α-bungarotoxin, Alexa-594 conjugate (B13423, 5 ug/mL, ThermoFisher), rabbit anti-Gap43 (NB300-143, 1:1000, Novus Biologicals), rabbit anti-SCG10 (NBP1-49461, 1:5000, Novus Biologicals), DAPI (D9542, 1:1000, Sigma) and fluorescence-conjugated secondary antibodies (1:1000, Jackson ImmunoResearch).
Validation	Antibodies meet all of the quality control standards defined by manufactures and validated by previous publications (Farrell and O'Farrell 2013). Validation statements for the commercial antibodies are available on the manufactures websites.

Eukaryotic cell lines

Policy information about cell lines

Cell line source(s)	The PIEZO1KO (5E3) HEK293T cells were from the Ardem Patapoutian lab. WT HEK293T cells were from ATCC.
Authentication	The cell line was confirmed by phenotypic analyses.
Mycoplasma contamination	All cell lines tested negative for mycoplasma contamination.
Commonly misidentified lines (See ICLAC register)	No commonly misidentified cell lines were used in the study.

Animals and other organisms

Policy information about studies involving animals; ARRIVE guidelines recommended for reporting animal research

Laboratory animals	Drosophila melanogaster is used in the study. Both male and female flies are collected at the third instar larval stage. Mouse (postnatal day 0-1) and rat embryos (embryonic day 17-18) are used for cell culture. Mice adults (4-8 weeks old) of both sexes are

	also used. The genotypes are described in the main text and Methods.
Wild animals	No wild animals were used in the study.
Field-collected samples	No field collected samples were used in the study.
Ethics oversight	All studies and procedures involving animal subjects were performed under the approval of the Institutional Animal Care and Use Committee (IACUC) at the Children's Hospital of Philadelphia and Temple University.

Note that full information on the approval of the study protocol must also be provided in the manuscript.
